# Illustrations of interactions needed when investigating sleep using a type of AM-PM PM-AM design

**DOI:** 10.3758/s13423-023-02248-8

**Published:** 2023-06-15

**Authors:** Laura Mickes, David P. Morgan, Darío A. Fuentes Grandón, Stewart Boogert, Nina Kazanina

**Affiliations:** 1 University of Bristol, Bristol, UK; 2 Department of Clinical Psychology, Heidelberg University, Heidelberg, Germany; 3 Department of Addiction Behaviour and Addiction Medicine, Heidelberg University, Heidelberg, Germany; 4 Department of Psychiatry and Psychotherapy, Medical Faculty Mannheim, Central Institute of Mental Health, Heidelberg University, Heidelberg, Germany; 5 Royal Holloway, University of London, London, UK

**Keywords:** Time-of-day controls, Circadian rhythm controls, 2 × 2 interactions, Unequal variance signal detection

## Abstract

Sleep has long been thought of and promoted to be beneficial for memory. Some claims that sleep aids memory have been made in the absence of a critical interaction. This condition is necessary when using a commonly-used experimental design (a type of AM-PM PM-AM design). We propose that a sleep effect exists only if there is an interaction between groups (experimental and time-of-day controls) and the time of test or study (morning and evening). We show different patterns of results that would and would not support a sleep effect with empirical and model-generated data from recognition memory experiments and hypothetical data. While we use these data to make our point, our suggestions apply to any memory and non-memory-related investigation (e.g., emotional memory, false memory susceptibility, language learning, problem-solving). Testing for and finding the proper interaction will add to the evidence that sleep boosts performance.

**Supplementary Information:**

The online version contains supplementary material available at 10.3758/s13423-023-02248-8.

Papers on the topic of sleep and cognition, especially memory, often include emphatic claims about the dramatic benefits of sleep (e.g., Ashton et al., 2019; Feld et al., 2013; Huguet et al., 2019; Inostroza & Born, 2013; Morgan et al., 2019; Rasch & Born, 2013; but see Ackermann et al., 2015; Cordi & Rasch, 2021). In a review with 1.6 k citations, including 385 recent ones, according to the journal’s article metrics (August 2022), the authors wrote, “Over more than a century of research has established the fact that sleep benefits the retention of memory” (Rasch & Born, 2013, p. 681). These conclusions are made based on results from studies that use one of many experimental designs. We suggest that if a certain design (AM-PM PM-AM) is used, to support the claim that sleep aids memory or other cognitions, there must be a significant interaction between experimental and control groups and AM and PM test (or study) times. It is rare to report this interaction in the literature, and it needs to be to show that sleep does indeed benefit memory, or whatever the phenomenon under investigation is.

While we focus on recognition memory in this paper, our recommendations apply to any investigation of sleep’s impact, including recall memory, autobiographical memory, associative memory, emotional memory, false memory susceptibility, language learning, motor learning, creativity, and problem-solving. We show the patterns of results that would support the claim that sleep benefits memory with experimental, simulated, and hypothetical data. As our point is a methodological one, we stay atheoretical when referring to a sleep benefit, which, according to various accounts, could be due to boosted consolidation, protection from interference, or contributions of both (for reviews on prominent theories, see Berres & Erdfelder, 2021; Cordi & Rasch, 2021; Squire et al., 2015; Wixted, 2005).

Several different experimental designs aim to understand sleep’s effects, including pharmacological manipulations (e.g., Feld & Diekelmann, 2015; Pinggal et al., 2022), stimulation methods (e.g., Cellini & Mednick, 2019; Grimaldi et al., 2020), and targeted memory reactivation (e.g., Cellini & Capuozzo, 2018; Hu et al., 2020). In many naturalistic sleep experiments, there is a daytime wake group (AM-PM) and a night-time sleep group (PM-AM). In investigations of sleep and recognition memory, participants study a list of stimuli and test on the same “old” stimuli (targets) and “new” items (lures). When the study and test phases occur differ for the different groups. Participants in the wake group take part in the study phase in the morning (AM) and, about 12 h later, are tested in the evening (PM) after going about their days. Participants in the sleep group take part in the study phase in the evening (PM) and, about 12 h later, are tested in the morning (AM) after sleeping. If memory is better for the sleep group, then sleep is thought to be beneficial for memory (e.g., Jones et al., 2018; Wilhelm et al., 2011).

Time-of-day effects can masquerade as sleep benefits because performance, including memory performance, fluctuates throughout the day (e.g., Barrett & Ekstrand, 1972; Folkard & Monk, 1980; Keisler et al., 2007; Nesca & Koulack, 1994; Pan & Rickard, 2015; Schmidt et al., 2007). Most articles involving naturalistic sleep experiments include concerns about time-of-day, or circadian, effects and at least one of many ways to mitigate the potential impact. Some designs, about 20% in a recent large metanalytic review paper (Berres & Erdfelder, 2021), are within-subject designs where participants take part in both sleep and wake groups (e.g., Gais et al., 2006; Maski et al., 2015; Prehn-Kristensen et al., 2009; Schönauer et al., 2015).

The other 80% are between-subjects studies. Many include analyses comparing groups' difference scores (memory performance on the later testing is subtracted from performance immediately after learning the material; e.g., Nissen et al., 2011; Sonni & Spencer, 2015; van Rijn et al., 2017). Other approaches include comparing outcomes on various scales, including sleepiness scores (e.g., Diekelmann et al., 2010; van Rijn et al., 2017) and mood scales (e.g., Prehn-Kristensen et al., 2015), and other cognitive tests (e.g., working memory tests; Potkin & Bunney, 2012). Still, others use data from nap studies as a control (e.g., Baran et al., 2010; Payne et al., 2009). Some researchers collect data from participants with 24-h retention intervals (Ellenbogen et al., 2006). Some combine multiple approaches (e.g., Diekelmann et al., 2010; Himmer et al., 2017; Jurewicz et al., 2016; Kurdziel & Spencer, 2016). And some suggest follow-up research is needed that controls for time-of-day effects (e.g., Hupbach, 2018; Takeuchi et al., 2014). Any methodological concerns about these approaches to cope with the challenging time-of-day problem are outside the scope of this paper (see Nemeth et al., 2021). The ever-present concern for time-of-day confounds has led to varied attempts to control them. We present an improvement to one approach (that could be applied more widely).

Using time-of-day control participants is another way researchers deal with the potential confound (e.g., Abel & Bäuml, 2012, 2013; Bäuml et al., 2014 Experiments 1 and 3; Baran et al., 2012; Bennion et al., 2015; Bennion et al., 2017; Chambers & Payne, 2014; Ekstrand, 1967; Fenn et al., 2009; Lutz et al., 2017; Monaghan et al., 2015; Morgan et al., 2019; Payne et al., 2008; Racsmány et al., 2010, Experiment 2; Scullin & McDaniel, 2010; Sheth et al., 2009). The inclusion of the control groups is a prerequisite for the analysis we recommend. Unlike the experimental groups with approximately 12-h long wake- or sleep-filled retention intervals, the control groups participate in one session, with considerably shorter retention intervals. Participants in the AM control group take part in the study and test phases in the morning, and participants in the PM control group take part in the study and test phases in the evening. If performance is better for the AM control group than the PM control group, and if performance is better for the sleep group than the wake group, this could reflect that testing in the AM benefits memory, not that sleep, per se, benefits memory. That is, performance could be better because the sleep group and AM control group take the test in the morning.^1^ This may be the case even if pairwise comparisons are made and the difference in accuracy is significant for the experimental groups but not for the control groups. Assuming this pattern of results supports a sleep benefit is, in effect, turning the null hypothesis of no difference between the control groups into a finding (Gelman & Stern, 2006). If there is no interaction of performance between the groups and the test time, then it is a mistake to claim a sleep benefit. The results need to show that having a period of sleep between the study and test phases improves memory over a period of wake, beyond being tested in the morning. These analyses, however, are not reported in the literature, with one exception (Scullin & McDaniel, 2010).

Instead of conducting the analysis that we suggest, it is most common to analyze the data from the experimental and control groups separately (e.g., Baran et al., 2012; Bäuml et al., 2014 Experiments 1 and 3; Bennion et al., 2015; Bennion et al., 2017; Chambers & Payne, 2014; Ekstrand, 1967; Fenn et al., 2009; Monaghan et al., 2015). These experiments set a precedent for research that follows, including ours. We published a registered report in which the first stage included our analysis plans (Morgan et al., 2019). We proposed to use the standard AM-PM PM-AM design with time-of-day controls. The planned analyses included separately analyzing the experimental and control data. In the Stage 1 manuscript, we wrote that we would rule out a time of day confound by simply comparing the AM control group to the PM control group. Our implicit assumption was that there would be no difference between the AM and PM control groups. Only after the results were published did we realize this is the wrong approach to support a true sleep benefit.^2^

The right approach is to test for the right interaction. There is not enough information reported in the literature to test for the interaction with published data. We contacted the corresponding authors of the experiments that were set up in a way allowing us to conduct the recommended analysis. We received data from approximately 30% of our requests, resulting in data from multiple experiments collected by one lab and three experiments from two other labs (including the current paper’s corresponding author’s lab). Even in the limited number of datasets, we found half of the recall data yielded a significant interaction, thus providing existence proof that the method here has utility. We offer empirical and simulated data (and hypothetical data in the discussion section) as examples of interactions that would and would not support that memory is better when sleep occurs after learning.

## Empirical data

The empirical data are from a standard list-learning recognition memory experiment in which we used the AM-PM PM-AM design with time-of-day control groups. There were two lists of English (List 1) and Japanese (List 2) words for participants to try to remember. As we collected the data for illustrative purposes to make our methodological point, not to test a hypothesis, we did not choose a sample size in advance.^3^ We also use these data to generate simulated data to provide more examples.

## Method

### Participants

Participants (*N* = 178) were first-year students enrolled in the psychology undergraduate program at the University of Bristol. All participants consented to take part. The University of Bristol Ethics Board gave ethical approval, #030,321,116,988. Due to a computer issue, data from one participant were excluded from the analyses. Another participant gave the same response to all items, so those data were also excluded from all analyses.

Group assignment depended on whether participants were born on an odd or even day and month. This assignment yielded 46 participants in the AM control group, 58 participants in the PM control group, 39 participants in the wake group, and 33 participants in the sleep group (138 female, 38 male; *µ*_age_ = 19.82 years, *SD* = 2.58). Ages were not significantly different by group, *F*(3, 172) = 0.631, *p* = 0.596. Because nine participants indicated knowing some Japanese, their data were excluded from List 2 analyses. Without those participants, there were 45 participants in the AM control group, 53 participants in the PM control group, 39 participants in the wake group, and 31 participants in the sleep group (132 female, 36 male, *µ*_age_ = 19.79 years, *SD* = 2.53). Ages were not significantly different by group, *F*(3, 164) = 0.686, *p* = 0.562.

### Materials

#### Stimuli

List 1 was made of 50 English monosyllabic concrete nouns (e.g., *girl*, *tent*), half animate and half inanimate and List 2 was made of 50 Japanese nouns (e.g., *tori*, *nezumi*), half bisyllabic and half trisyllabic. For both lists, we randomly selected 25 words as targets and 25 words as lures, so that there was an approximately equal proportion of animate versus inanimate words (List 1) and two- versus three-syllable words (List 2) for targets and lures. Eight practice words (four words per list) were not on List 1 or List 2.

#### Sleep-related information

We administered the Epworth Sleepiness Scale, which measures general daytime sleepiness (Johns, 1991). We also asked participants how many hours they slept the night prior to the day they took part in the experiment. Participants in the sleep group reported hours they slept for 2 nights.

#### Individual information

Participants provided their age and sex, and whether they speak Japanese, to what extent, and if they knew the meaning of any of the words on List 2.

### Procedure

All participation took part online using the platform, Gorilla (Anwyl-Irvine et al., 2020). Participants in the control groups completed the experiment in one session. Participants in the AM control group completed the experiment between 8 a.m. and 11 a.m., and participants in the PM control group completed the experiment between 8 p.m. and 11 p.m. Participants in the experimental groups completed the experiment in two sessions. The first session was the study phase, and the second session was the test phase. Participants in the wake group completed the first session between 8 a.m. and 11 a.m. and completed the second session on the same day between 8 p.m. and 11 p.m. Participants in the sleep group completed the first session between 8 p.m. and 11 p.m. and completed the second session the following morning between 8 a.m. and 11 a.m. (Himmer et al., 2017; Morgan et al., 2019). Whether they participated in one or two sessions, the experiment took the same overall time.

After consenting, the participants proceeded with the study phase for List 1 (English words) followed by List 2 (Japanese words). They were instructed that they would see English and Japanese words and that their memory for the words would be tested later. For List 1, participants had 3 s to indicate with key presses whether each word corresponded to an animate or inanimate object. For List 2, participants had 3 s to indicate with key presses whether each word contained two or three syllables. Between items, a fixation cross appeared for 250 ms. Each list was preceded by a 4-item practice session which was identical to the task except overall feedback was provided (e.g., “You were correct on 3 out of 4 trials.”).

During the test phase, participants indicated whether each item was presented during the study phase on a 6-point scale (*100% certain not on the list*, *probably not on the list*, *maybe not on the list*, *maybe on the list*, *probably on the list*, *100% certain on the list*). Responses were self-paced. All items presented during the practice, study phase, and test phase were randomly presented.

At the end of the experiment, participants answered the Epworth Sleepiness Scale questions (Johns, 1991), reported how many hours they slept the night before, and answered the demographic questions. They were debriefed later during class.

## Results

### Empirical data

Here we focus on the results that directly bear on our point about interactions, and therefore provide the Epworth Sleepiness Scale scores and sleep time comparisons across groups in Supplementary Information. The data and word lists are available at the Open Science Framework (https://osf.io/vmjry/?view_only=8f68a6f60fd44ec4817f983e9d3a8e1e).

To measure discriminability (i.e., the ability to discern targets from lures), *d*_*a*_ scores were computed for each participant and then averaged across participants in each group. *d*_*a*_ is like *d′*, but it does not assume that the target and lure distributions have equal variances and is often more appropriate for data from list-learning experiments (e.g., Mickes et al., 2007; Rotello et al., 2008). The dependent variable *d*_*a*_ is given by

1 $${d}_{a}={\left(\frac{2}{1+{s}^{2}}\right)}^\frac{1}{2}\left(z\right.(H)-s z(F)),$$

where *z(H)* and *z(F)* are the z-scores of hit and false alarm rates, respectively, and *s* is the *z*-ROC slope (Macmillan & Creelman, 2005). The slope gives an estimate of σ_lure_/σ_target_. We used 0.80 for all *z*-ROC slopes.

**Fig. 1:**
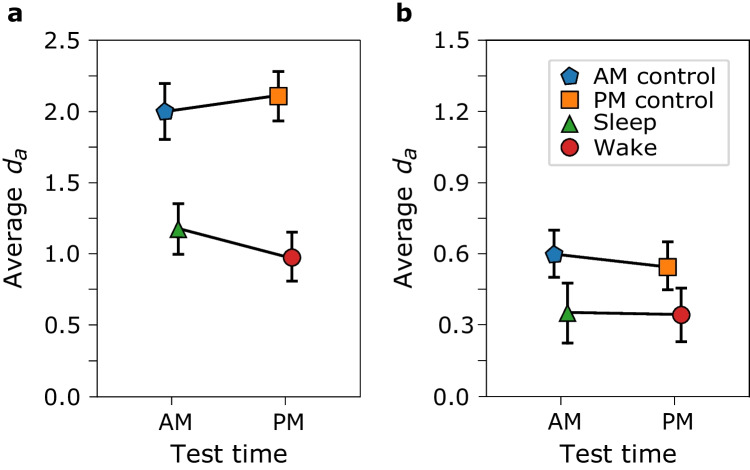
Between-subjects 2 × 2 ANOVA. Average *d*_*a*_ by group as a function of time of test for List 1 (**a**) and List 2 (**b**). The error bars are 95% confidence intervals. The different colors and shapes represent the different groups. The interactions were not significant. (Colour figure online)

We used JASP for conducting inferential statistical tests (JASP team, 2022). We performed a 2 (group: experimental vs. control) × (time of test: morning vs. evening) ANOVA on the *d*_*a*_ values. Figure 1a and b shows the average *d*_*a*_ scores by group as a function of test time for List 1 and List 2, respectively.

Table 1 shows the ANOVA results. There was not a significant main effect of time of test (morning vs. evening), there was a significant main effect of group (experimental: wake/sleep vs. control: AM/PM), and the interaction was not significant for List 1 or List 2. The main effect of group is expected given that the control groups’ retention interval is considerably shorter than the experimental groups’ retention interval.

**Table 1:** Empirical data ANOVA results for List 1 and List 2

Type of comparison	*df*	*F*	*p* value	*η*^*2*^
List 1				
Time of test	1/172	0.234	0.629	< 0.001
Group	1/172	107.64	< 0.001	0.381
Interaction	1/172	2.536	0.113	0.009
List 2				
Time of test	1/163	0.291	0.590	0.002
Group	1/163	17.537	< 0.001	0.097
Interaction	1/163	0.161	0.688	< 0.001

### Model fits and simulated data

**Fig. 2:**
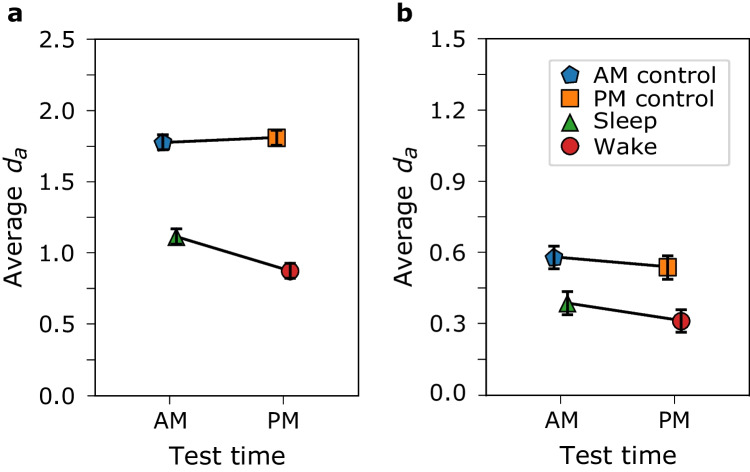
Between-subjects 2 × 2 ANOVA. Average *d*_*a*_ by group as a function of time of test for the simulated data from List 1 (**a**) and List 2 (**b**). The different colors and shapes represent the different groups. The error bars are 95% confidence intervals. The interaction is significant in **a** but not in **b**. (Colour figure online)

In the empirical data, the interactions were not significant. There may not have been enough power in our opportunity sample to detect a small interaction effect. Our goal with data collection was to provide examples and to use them for generating data. We generated data using the unequal variance signal detection model (UVSD; Egan, 1958; Ratcliff et al., 1992; Wixted, 2007) fitted to the empirical data. The details of the fits and data generation are presented in the Supplementary Information. The average *d*_*a*_ values of the simulated data are shown in Fig. 2.

As with the empirical data, the *d*_*a*_ values from the generated data were subjected to a 2 (group: experimental vs. control) × 2 (time of test: morning vs. evening) ANOVA test. The results are shown in Table 2. There was a significant, small interaction in the List 1 simulated data. This interaction is needed to claim that sleep benefits memory. The interaction was not significant in the List 2 simulated data. List 2 data provide an opportunity to show the pattern of results that can lure researchers into erroneously concluding a sleep benefit. That is, the sleep group significantly outperforms the wake group but the difference between the control groups is not significantly different.

**Table 2:** Simulated data ANOVA results for List 1 and List 2. The interaction values are in bold

Type of comparison	*df*	*F*	*p* value	*η*^*2*^
List 1				
Time of test	1/756	14.71	< 0.001	0.009
Group	1/756	886.59	< 0.001	0.527
Interaction	**1/756**	**26.13**	** < 0.001**	**0.016**
List 2				
Time of test	1/756	6.05	0.014	0.007
Group	1/756	76.03	< 0.001	0.091
Interaction	1/756	0.50	0.482	< 0.001

To show how this pattern can be misleading, we conducted *t-*tests on the List 2 *d*_*a*_ scores from the experimental groups (sleep vs. wake) and control groups (AM vs. PM), as has been done in the sleep literature. Because the difference in *d*_*a*_ scores between the control groups was not significant, *t*(378) = 1.22, *p* = 0.223, but the difference between the experimental groups was significant, *t*(378) = 2.27, *p* = 0.024, one may be tempted to conclude a sleep benefit. However, the lack of the interaction from the ANOVA, *p* = 0.482, does not support a sleep benefit interpretation. Moreover, if time-of-day controls were not included, then the significant difference in scores between the sleep group and wake group would be erroneously interpreted as a sleep benefit.

## Discussion

We propose that interactions are needed to strengthen claims about sleep’s benefits on memory when using time-of-day control groups with experimental groups using the specific design described here. We presented empirical and simulated data, showing one acceptable interaction with the List 1 simulated data. However, not all interactions are sufficient (e.g., Pashler et al., 2008; Redick, 2015).

**Fig. 3:**
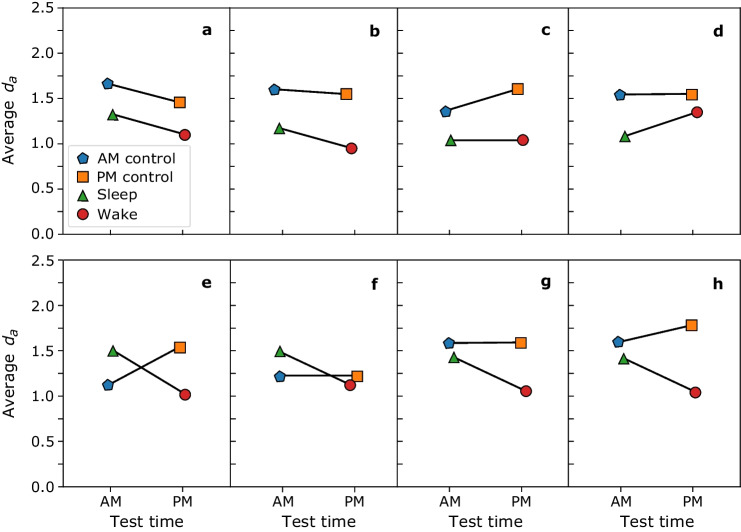
Between-subjects 2 × 2 ANOVA for eight hypothetical datasets. Average *d*_*a*_ by group as a function of time of test. The different colors and shapes represent the different groups. There are no acceptable patterns in the top panels, **a-d**, and there are acceptable interactions in the bottom panels, **e–h**. (Colour figure online)

Figure 3 shows hypothetical data plotted in different patterns, including acceptable and unacceptable interactions, to demonstrate that the interaction must take a specific form to support a sleep benefit. Consider that these hypothetical data are from the same type of recognition memory experiment presented earlier. Figure 3 illustrates eight possible outcomes of this hypothetical experiment. As in Figs. 1 and 2, in each panel, *d*_*a*_ is plotted by group (experimental and control) as a function of test time (morning or evening).

In the top panels, Fig. 3a–d, no patterns would support a sleep benefit claim. In Fig. 3a, the sleep group outperformed the wake group, and the AM control group outperformed the PM control group. Thus, the groups tested in the morning performed better than those tested in the evening. In Fig. 3b, the AM control group performed better than the PM control group, as did the sleep group over the wake group. Yet neither difference is enough for a significant interaction. The pattern of the experimental group data in Fig. 3a and b may be confused for a sleep benefit without considering the AM and PM control group data. However, the lack of the interaction shows that would be the wrong conclusion.

Figure 3c shows the PM control group outperformed the AM control group, but the experimental groups performed similarly. The pattern, therefore, supports a time-of-day effect, not a sleep one. Figure 3d shows an interaction in the opposite direction where the wake group outperforms the sleep group. The lack of significant interactions in Fig. 3a and b and the interactions in Fig. 3c and d in the wrong direction precludes interpretations of a sleep benefit.

In the bottom panels, Fig. 3e–h, the interactions allow for interpretations of a sleep benefit. In Fig. 3e and f, the cross-over interactions are evident. In Fig. 3e, the sleep group performed better than the wake group, and the AM control group performed worse than the PM control group. This pattern clearly shows the interaction and that both sleep and time of day contributed. Similarly, in Fig. 3f, the sleep group outperformed the wake group, and the am and pm groups performed alike. Thus, the interaction is driven by the experiment groups’ performance. The patterns in these two figures (Fig. 3e and f) are unlikely given that the control groups have a substantially shorter retention interval than the experimental groups. While unlikely, that pattern is not unheard of (e.g., Abel & Bäuml, 2013), and it is a type of acceptable interaction.

In Fig. 3g, the sleep group outperformed the wake group, and the control groups’ performance is similar. Therefore, the experimental groups drove the interaction. The patterns in Fig. 3f and g clearly show that the time the test was taken did not influence the interaction. Figure 3h is similar, where the sleep group outperformed the wake group, but the PM control group outperformed the AM control group. This pattern indicates sleep and time-of-day influenced performance, both driving the interaction. All the interactions in Fig. 3e–h allow for interpretations of a sleep benefit. Note that the data can be plotted the other way with the group on the* x*-axis and the test time as separate lines and it may be easier to see a crossover interaction this way.

Interpreting interactions is challenging (e.g., Redick, 2015; Rohrer & Arslan, 2021). Because *d*_*a*_ (and *d'*) theoretically lies on an interval measurement scale, given the assumptions of signal detection theory, interactions can be meaningfully interpreted (Embretson, 1996). A signal detection-based theory is a theory of underlying memory signals, with a firm grounding in measurement methodology dating back to Fechner (Fechner 1860/1966; see Wixted, 2019, for a historical review). According to Wickelgren (1974),

Using the methods of statistical decision theory, an interval-scale measurement of the memory strength is possible for recognition memory, making only rather weak and plausible assumptions concerning the decision process that translates strength into yes-no decisions (p. 776).

Thus, if the theory embraced underlying *d*_*a*_ (or *d'*) is signal detection-based, there is further credence for interpreting any interactions.

This brings us to an important caveat regarding other kinds of dependent measures. Non-crossover interactions can be changed by transformations of the data such that there is no longer an interaction and if there is no noncrossover interaction, transformations of the data can change that outcome into an interaction (Loftus, 1978; Wagenmakers et al., 2012). This feature applies when memory is tested with a recall test yielding proportion correct scores subjected to an interaction test. Unlike when *d*_*a*_ (or *d'*) is considered, it is highly unlikely that there is a linear relationship, or direct mapping, between the dependent measure (i.e., the proportion of items correctly recalled) and the underlying memory processes (i.e., recall; Wixted, 1990). Therefore, any interpretation of a noncrossover interaction test of proportion correct scores, even if statistically significant, should be made with caution.

The methodological limitations include unequal sample sizes. Unequal group sizes may decrease the ability to detect an interaction. However, reducing the sample size to match the group with the smallest *n* yielded the same results—no significant interactions—as with the full sample size. Another limitation is that our opportunity sample of university students may not reflect a wider demographic (e.g., Schlarb et al., 2017). Yet another limitation is the varied and large window of time for participation. Presenting the stimuli closer to sleep would reduce interference in the sleep group.

Going forward, new experiments should be designed to allow for the test of the interaction. If future empirical results turn out such that there is a critical interaction, as there was in the List 1 simulated data, then an interpretation of a sleep benefit can be made. If, on the other hand, there is no interaction, despite there being significantly better performance in the sleep group than the wake group but no significant difference between the control group's performance, as there was in the List 2 simulated data, no sleep benefit claims should be made. Ideally, the robustness of the memory benefit when memory is probed by recall and recognition tests is established before drilling down into smaller components (emotional vs. nonemotional, feedback vs. no feedback; recollection vs. familiarity, etc.). Additionally, the interaction in the behavioral data must exist before making any links to the physiological or neural data (Krakauer et al., 2017).

Our focus on one type of design does not minimize our point as researchers use the results from these studies to support the idea that there is a sleep benefit. The data from these studies are also included in meta-analyses that provide overviews of the state of the field (e.g., Berres & Erdfelder, 2021; Lipinska et al., 2019; Schäfer et al., 2020). There are a host of designs to understand the impacts of sleep. This between-subjects design is a convenient way to measure sleep’s effects, necessary for some investigations, and when used, interactions are key. Implementing this analysis and meaningfully interpreting the results would mean making some changes to research practices (see also Nemeth et al., 2021). Unambiguously finding the critical interaction is one way to minimize concerns about a time-of-day confound and help determine if memory is better when we sleep after learning compared to a retention interval filled with daytime wakefulness.

### Supplementary Information

Below is the link to the electronic supplementary material.

Supplementary file1 (DOCX 151 KB)

## Footnotes

1 We are agnostic about whether study or test time is used for the purposes here. We opted for the time of test over time of study because of the evidence that time of test, not sleep or time of study, boosts motor learning outcomes (Pan & Rickard, 2015). It may not be the case for declarative memory, and these are interesting future considerations.

2 Fortunately, the ideas proposed in the current paper do not change the conclusions reported by Morgan et al. (2019).

3 Our sample size of 176 is larger than most of the studies, where, according to a recent meta-analysis, the median sample size was 16 participants per group (Berres & Erdfelder, 2021).
