# Supplementary material for: Illustrations of interactions needed when investigating sleep using a type of AM-PM PM-AM design
Source: Psychon Bull Rev. 2023 Jun 15;30(6):2106–15. doi: 10.3758/s13423-023-02248-8 (PMC10728231; doi:10.3758/s13423-023-02248-8)
Supplement: Supplementary file 1 — Supplementary file1 (DOCX 151 KB) [file 13423_2023_2248_MOESM1_ESM.docx]

**Supplementary Information**

**Results**

The results pertaining to our point about interactions are presented in the main article. For completeness, we present the analyses of the Epworth Sleepiness Scale scores and sleep times here in Supplementary Information (SI). The Epworth Sleepiness Scale scores for the AM control group (*µ* = 7.76, *SD* = 4.32), PM control group (*µ* = 8.68, *SD* = 3.88), sleep group (*µ* = 7.24, *SD* = 3.49), and wake group (*µ* = 6.67, *SD* = 3.12) were not significantly different, *F*(3,172) = 2.43, *p* = .067. Sleep time (i.e., time slept before the test), in minutes, for the AM control group (*µ* = 436.61, *SD* = 77.33), PM control group (*µ* = 456.04, *SD* = 94.90), sleep group (*µ* = 432.32, *SD* = 85.02), and wake group (*µ* = 448.53, *SD* = 72.90) was not significantly different either, *F*(3,168) = .75, *p* = .526. Four participants did not report sleep time.

**Model fits and simulated data**

Simulated data were generated using the unequal variance signal detection model (UVSD; Egan, 1958; Ratcliff et al., 1992; Wixted, 2007) fitted to the empirical data. SI Figure 1 shows the conceptualization of the UVSD model, in which a Gaussian distribution represents the memory strength of the lures, and another Gaussian distribution represents the memory strength of the targets. The top panel shows *µ*_l_ and σ_l_, which are the mean and standard deviation of the lure distribution, respectively, and *µ*_t_ and σ_t_ which are the mean and standard deviation of the target distribution, respectively. Respondents set a decision criterion along an axis of memory strength.

The bottom panel of SI Figure 1 shows the decision criteria, represented by c_1_, c_2_, c_3_, c_4_, c_5_, and c_6_, where c_6_ maps onto the highest level of confidence. If a memory signal generates a strength that is greater than the criterion c_6_, a participant will give a high confidence response that the item is old (i.e., the item was on the study list). If the memory signal strength exceeds the criterion c_5_, but not c_6_, the participant expresses that the item was probably on the study list, and so on. In empirical data, the standard deviation of the target distribution is often about 1.25 times that of the lure distribution (i.e., a *z*-ROC slope of .80; e.g., Mickes et al., 2007), which is why we used this model to generate simulated data.

SI Figure 1. Unequal variance signal detection model with a lure distribution with *µ*_l_ and σ_l_ and target distribution with *µ*_t_ and σ_t_ along a memory strength axis (top panel). The target distribution has greater variance than the lure distribution σ_t_ is typically 1.25 times greater than σ_l_). The bottom panel shows the different criterion c_1_, c_2_, c_3_, c_4_, c_5_, and c_6_).

We used pyWitness (https://lmickes.github.io/pyWitness/), a python toolkit for analyzing recognition memory data and conducting signal detection-based fits, to perform the model fits to generate the simulated data. For the UVSD model fits, lures (items that were not on the study list) were randomly drawn from the lure distribution, and targets (items that were on the study list) were randomly drawn from the target distribution. The lure parameters *µ*_lure_ and σ_lure_ were fixed at 0 and 1, respectively. The target parameters *µ*_target_ and σ_target_, criterion, and c_2_-c_6_ were free to vary. c_1_ does not vary because it includes all responses.

SI Figure 2 shows the empirical ROC curves for each group, and the corresponding UVSD fits. The ROC plots cumulative hit and false alarm rates pairs for every level of confidence. The further the points fall from the line of chance performance, the better able participants are at discriminating targets from lures. To estimate confidence intervals for the ROC points, participants were randomly sampled with replacement using a 200-replication bootstrap procedure.

The UVSD model was fit to the experimental data by minimizing χ^2^, calculated from the number of responses in each confidence bin. The *p*-values are calculated from the fit χ^2^ and the number of degrees of freedom. SI Table 1 shows the fit parameters of the UVSD model to the empirical data. As shown in SI Figure 2 and from the *p*-values of SI Table 1, the UVSD model is a good description of the data.

SI Figure 2. Receiver operating characteristic (ROC) curves for each group for List 1 (a) and List 2 (b). The corresponding model fits are represented by the dashed curves. The black dashed line represents chance performance. The point size differences reflect relative frequency. The error bars are 95% confidence intervals.

SI Table 1. Unequal variance signal detection model fit parameters for each group for List 1 and List 2

|  | AM control | PM control | Sleep | Wake |
| --- | --- | --- | --- | --- |
| List 1 |  |  |  |  |
| *µ*_l_ | 0 | 0 | 0 | 0 |
| σ_l_ | 1.00 | 1.00 | 1.00 | 1.00 |
| *µ*_t_ | 2.43 | 2.42 | 1.42 | 1.02 |
| σ_t_ | 1.33 | 1.32 | 1.27 | 1.17 |
| *c_1_* | -0.39 | -0.05 | -0.84 | -0.76 |
| *c_2_* | 0.29 | 0.42 | -0.10 | 0.02 |
| *c_3_* | 0.80 | 0.79 | 0.49 | 0.47 |
| *c_4_* | 1.27 | 1.13 | 0.97 | 0.88 |
| *c_5_* | 1.74 | 1.51 | 1.53 | 1.42 |
| ndf | 4 | 4 | 4 | 4 |
| *χ^2^* | 4.85 | 7.92 | 3.83 | 5.47 |
| *p*-value | 0.303 | 0.095 | 0.430 | 0.242 |
| List 2 |  |  |  |  |
| *µ*_l_ | 0 | 0 | 0 | 0 |
| σ_l_ | 1.00 | 1.00 | 1.00 | 1.00 |
| *µ*_t_ | 0.66 | 0.58 | 0.43 | 0.35 |
| σ_t_ | 1.07 | 1.07 | 1.12 | 1.02 |
| *c_1_* | -1.14 | -0.92 | -1.40 | -1.11 |
| *c_2_* | -0.41 | -0.27 | -0.52 | -0.41 |
| *c_3_* | 0.17 | 0.13 | 0.12 | 0.21 |
| *c_4_* | 0.61 | 0.56 | 0.62 | 0.70 |
| *c_5_* | 1.14 | 1.09 | 1.18 | 1.36 |
| ndf | 4 | 4 | 4 | 4 |
| *χ^2^* | 1.82 | 10.65 | 15.69 | 4.12 |
| *p*-value | 0.768 | 0.031 | 0.003 | 0.390 |

ndf = number of degrees of freedom.

We used the UVSD model fits to generate simulated data with an arbitrary number of participants and trials per participant. The simulated data were generated with 50 trials for each of the 190 participants per group based on a power analysis. The power analysis was based on data from a highly cited paper (Baran et al., 2012), where the sleep group significantly outperformed the wake group, but the AM and PM groups did not significantly differ. To have 90% power to detect a small interaction effect size of .03, we would need to collect data from 190 participants per group, 760 in total (Lakens & Caldwell, 2019). These values were obtained by computing *d'* values from Table 2 of Baran et al. for the neutral stimuli.

**References**

Egan J (1958). Recognition memory and the operating characteristic Tech. Note AFCRC-TN-58-51, (Bloomington: Indiana University, Hearing and Communication Laboratory), Technical report.

Johns MW (1991) A new method for measuring daytime sleepiness: The Epworth sleepiness scale. *Sleep 14(6),* 540–545.

Lakens D, & Caldwell A (2019) Simulation-Based Power-Analysis for Factorial ANOVA Designs. *Advances in Methods and Practices in Psychological Science*, 1–11.

Mickes L, Wixted JT, & Wais PE (2007) A direct test of the unequal-variance signal detection model of recognition memory. *Psychonomic Bulletin and Review 14(5),*858–865.

Ratcliff R, Sheu CF, & Gronlund SD (1992) Testing Global Memory Models Using ROC Curves. *Psychological Review 99(3),* 518–535.

Wixted JT (2007). Dual-process theory and signal-detection theory of recognition memory. *Psychological Review 114*(1), 152–176.
